# A Novel High-Mannose Specific Lectin from the Green Alga *Halimeda renschii* Exhibits a Potent Anti-Influenza Virus Activity through High-Affinity Binding to the Viral Hemagglutinin

**DOI:** 10.3390/md15080255

**Published:** 2017-08-16

**Authors:** Jinmin Mu, Makoto Hirayama, Yuichiro Sato, Kinjiro Morimoto, Kanji Hori

**Affiliations:** 1 Graduate School of Biosphere Science, Hiroshima University, Kagamiyama 1-4-4, Higashi-Hiroshima 739-8528, Japan; d134638@hiroshima-u.ac.jp (J.M.); hirayama@hiroshima-u.ac.jp (M.H.); 2 Faculty of Pharmacy, Yasuda Women’s University, Yasuhigashi 6-13-1, Asaminami-Ku, Hiroshima 731-0153, Japan; sato-y@yasuda-u.ac.jp (Y.S.); mori-k@yasuda-u.ac.jp (K.M.)

**Keywords:** lectin, green alga, *Halimeda renschii*, high-mannose specificity, anti-influenza virus activity

## Abstract

We have isolated a novel lectin, named HRL40 from the green alga *Halimeda renschii*. In hemagglutination-inhibition test and oligosaccharide-binding experiment with 29 pyridylaminated oligosaccharides, HRL40 exhibited a strict binding specificity for high-mannose *N*-glycans having an exposed (α1-3) mannose residue in the D2 arm of branched mannosides, and did not have an affinity for monosaccharides and other oligosaccharides examined, including complex *N*-glycans, an *N*-glycan core pentasaccharide, and oligosaccharides from glycolipids. The carbohydrate binding profile of HRL40 resembled those of Type I high-mannose specific antiviral algal lectins, or the *Oscillatoria agardhii* agglutinin (OAA) family, which were previously isolated from red algae and a blue-green alga (cyanobacterium). HRL40 potently inhibited the infection of influenza virus (A/H3N2/Udorn/72) into NCI-H292 cells with half-maximal effective dose (ED_50_) of 2.45 nM through high-affinity binding to a viral envelope hemagglutinin (K_D_, 3.69 × 10^−11^ M). HRL40 consisted of two isolectins (HRL40-1 and HRL40-2), which could be separated by reverse-phase HPLC. Both isolectins had the same molecular weight of 46,564 Da and were a disulfide -linked tetrameric protein of a 11,641 Da polypeptide containing at least 13 half-cystines. Thus, HRL40, which is the first Type I high-mannose specific antiviral lectin from the green alga, had the same carbohydrate binding specificity as the OAA family, but a molecular structure distinct from the family.

## 1. Introduction

Lectins are bioactive proteins of non-immune origin that recognize diverse sugar moieties in a non-catalytic manner. These proteins have been found in a wide range of organisms in nature from viruses to animals and function as recognition molecules in many biological processes, such as host–pathogen interaction, migration of lymphocytes, and cell communication. Studies on lectins have mainly been performed for land plants and animals from the viewpoints of their physiological functions and applications as convenient tools in glycomics because of their capability to discriminate the difference in carbohydrate structures. Practically, a number of lectins have been isolated and characterized from land plants and animals, some of which are now commercially available and are even employed in clinical applications including carbohydrate profiling, blood typing, cancer diagnoses, karyotyping, and assessing the immunocompetence of patients [1].

In recent decades, the molecular structures, oligosaccharide-binding specificity, and biological activities of hemagglutinins from marine algae (Rhodophyta and Chlorophyta) have extensively been studied and have demonstrated that algae may be a good source for novel and useful lectins because of their unique and algae-inherent properties. These recent investigations certainly revealed that algal lectins show strict binding specificity for some carbohydrate structures with novel molecular structures [2,3,4,5,6,7,8,9,10,11,12,13]. Meanwhile, high-mannose *N*-glycan (HM)-specific antiviral algal lectins, including those from blue-green algae (Cyanophyta) which are prokaryotes, have been attracting considerable attention owing to their prevention of virus infection with half-maximal effective concentration (EC_50_) in a low nanomolar range by blocking the entry of viruses, such as influenza viruses and human immunodeficiency viruses (HIV), to host target cells through their binding to the glycans on viral envelopes. These include the lectins from marine algae, *Griffithsia* sp. (Griffithsin or GRFT) [4], *Boodlea coacta* (BCA) [8], *Kappaphycus alvarezii* (KAA) [9,12], *Eucheuma serra* (ESA) [13] and from cyanobacteria, *Nostoc ellipsosporum* (Cyanovirin-N or CV-N) [14], *Oscillatoria agardhii* NIES-204 (OAA) [15], *Microcystis aeruginosa* PCC7806 (Microvirin or MVN) [16], *M. viridis* NIES-102 (MVL) [17], and *Scytonema varium* (Scytovirin or SVN) [18]. Although the antiviral activities of these algal and cyanobacterial lectins have mainly been examined against human immunodeficiency virus, some of them exhibit a broad range of antiviral activity against other viruses, including human influenza virus, Ebola virus, hepatitis C virus (HCV), Marburg virus (MARV), herpes simplex virus (HSV), and severe acute respiratory syndrome-corona virus (SARS-CoV) [19,20,21]. As for anti-influenza virus activity, cyanovirin-N [22] and a few high-mannose specific lectins from the marine algae—*Boodlea coacta* (BCA) [8], *K. alvarezii* (KAA-2) [9] and *E. serra* (ESA-2) [13]—were examined and found to possess the potent activity.

Human influenza virus, an enveloped virus containing a single-stranded segmented RNA genome, binds to the receptors possessing terminal sialic acids on the surfaces of epithelial cells through the viral envelope glycoprotein hemagglutinin [23,24]. The hemagglutinin is a heavily glycosylated viral surface protein, which guides the receptor recognition and virus entry to initiate the infectious process, carrying the *N*-linked glycans including HM type [23,24,25]. The main problem to fight against the influenza virus is the emergence of novel strains arising from the mutation [25,26], as well as in the case of anti-HIV [26]. Thus, the searches for an efficient vaccine having broad-spectrum activity against the diverse influenza virus strains are still ongoing. Several HM-specific algal and cyanobacterial lectins have been demonstrated to be endowed with potential anti-influenza virus activity through highly specific binding to the viral envelope hemagglutinin, although some of these proteins work in different oligosaccharide recognition mode as well as exhibit distinct primary structures [19,20,21,27]. This research suggests that the HM-specific algal lectins might be used as a novel strong antivirus agent against diverse virus strains possessing HMs on their envelopes.

During the survey for new useful lectins from marine algae in our laboratory, it was supposed that the marine green alga, *Halimeda renschii*, may contain an HM-specific lectin. However, there was no report to date on isolation of lectins from this alga. This situation motivated us to investigate the lectin from *H. renschii*. In this study, a novel HM-specific lectin from *H. renschii* was isolated and characterized.

## 2. Results

### 2.1. Purification of HM-Specific Lectin HRL40

**Figure 1:**
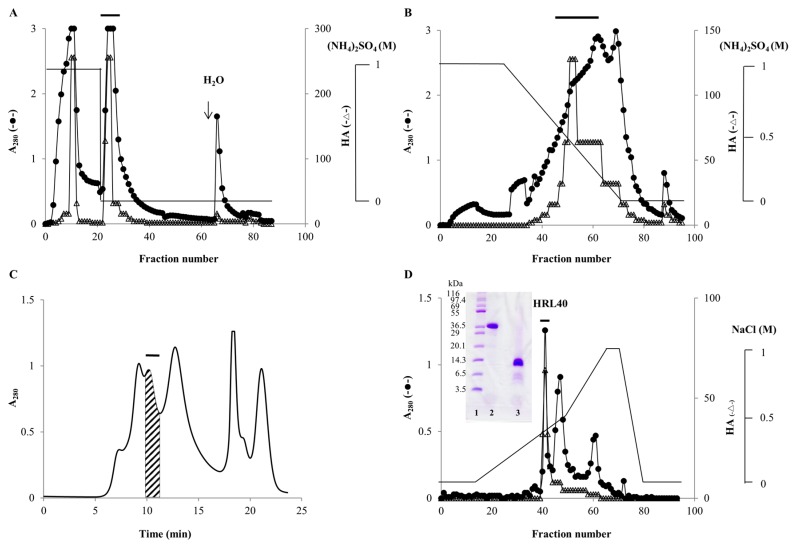
Purification of *Halimeda renschii* lectin (HRL40). (**A**) Hydrophobic chromatography with stepwise elution on a HiPrep Phenyl FF column (1.6 × 10 cm) of a 70% ammonium sulfate precipitate. Fractions of 10 mL were collected and measured for absorbance at 280 nm (A_280_) and for hemagglutination activity (HA). The active fractions eluted with 20 mM phosphate buffer (pH 7.0) (PB), which were denoted by a bar in the figure, were pooled for further purification; (**B**) Hydrophobic chromatography with a gradient elution (1.0–0 M) of ammonium sulfate in PB on the same HiPrep Phenyl FF column of the pooled active fractions obtained in panel A. The active fractions were pooled and concentrated by ultrafiltration; (**C**) Gel-filtration chromatography on a Superdex-75 column (1.0 × 30 cm) of the concentrated active fraction from panel B. Protein peaks were manually collected and the active protein peak denoted by a bar was collected; (**D**) Ion-exchange chromatography on a TSKgel DEAE-5PW column (0.75 × 7.5 cm) of the active peak obtained by gel-filtration. The active peak, denoted by a bar in the figure, was recovered as the purified lectin (HRL40). HRL40 was subjected to SDS-PAGE using a 12% polyacrylamide gel and after electrophoresis the gel was stained with Coomassie Brilliant Blue R-250 reagent. Lane 1, a molecular weight marker; lane 2, HRL40 treated without 2-mercaptoethanol; lane 3, HRL40 treated with 2% 2-mercaptoethanol (Figure 1D). In panels A, B and D, filled circles and open triangle show A_280_ and HA, respectively. Solid lines in panels A and B represent ammonium sulfate concentration in 20 mM phosphate buffer (pH 7.0), whereas that in panel D shows sodium chloride (NaCl) concentration in 20 mM Tris-HCl buffer (pH 8.0). The solid line in panel C represents the monitored A_280_.

From the powdered sample of *H. renshii*, lectin was extracted with 20 mM phosphate buffer (pH 7.0) (PB) containing 0.85% NaCl (PBS) and effectively recovered as a precipitate with 70% saturation of ammonium sulfate. In hydrophobic chromatography with stepwise elution of ammonium sulfate in PB, lectin was mostly adsorbed onto a HiPrep Phenyl FF column equilibrated with 1 M ammonium sulfate and then eluted with PB (Figure 1A). By re-chromatography of the non-adsorbed fraction, lectin was again recovered as PB-eluate in the same way. Both PB-eluates were combined and subjected to hydrophobic chromatography on the same column with a gradient elution (1.0–0 M) of ammonium sulfate in PB, in which the lectin was adsorbed and then eluted as a broad peak with 0.55–0.15 M ammonium sulfate (Figure 1B). The active peak fraction gave five major peaks in successive gel-filtration on a Superdex-75 column (Figure 1C). A major active peak obtained by gel-filtration was further purified by ion-exchange chromatography on a TSKgel DEAE-5PW column, affording a sharp protein peak with activity (Figure 1D). The purified lectin gave a single protein band of about 40 kDa and 10 kDa in non-reducing and reducing sodium dodecyl sulfate-polyacrylamide gel electrophoresis (SDS-PAGE), respectively (Figure 1D), and used for further experiments. The yield of the lectin, named HRL40 after the algal species *H. renschii* and its molecular weight, was 1.3 mg from 2 kg of the frozen alga (Table 1).

### 2.2. Hemagglutination-Inhibition Test

Carbohydrate binding specificity of HRL40 was first evaluated by hemagglutination-inhibition test with sugars and glycoproteins (Table 2). The hemagglutination activity (HA) of HRL40 was strongly inhibited by a few glycoproteins such as yeast mannan, porcine thyroglobulin (PTG), and asialo-PTG, all of which contain HM-glycans, but not by other glycoproteins bearing complex *N*-glycans and *O*-glycans, including transferrin, fetuin and mucin and their asialo-derivatives. All of monosaccharides and lactose examined were not inhibitory. From the results, HRL40 was suggested to have a binding preference for HM-glycans.

On the other hand, the HA of the crude lectin, such as salting-out fraction and PB-eluate in hydrophobic chromatography (stepwise elution), was not inhibited even in the presence of yeast mannan (Table 2), while that of the pooled active fractions, which was obtained by successive hydrophobic chromatography with a gradient elution of ammonium sulfate, was strongly inhibited by yeast mannan as well as the purified lectin HRL40 (Table 2). The intriguing results imply that there may exist some endogenous inhibitor(s) for HRL40 in the algal tissue, which could be separated from the lectin component by hydrophobic chromatography with a gradient elution of ammonium sulfate. 

### 2.3. Oligosaccharide-Binding Specificity of HRL40

**Figure 2:**
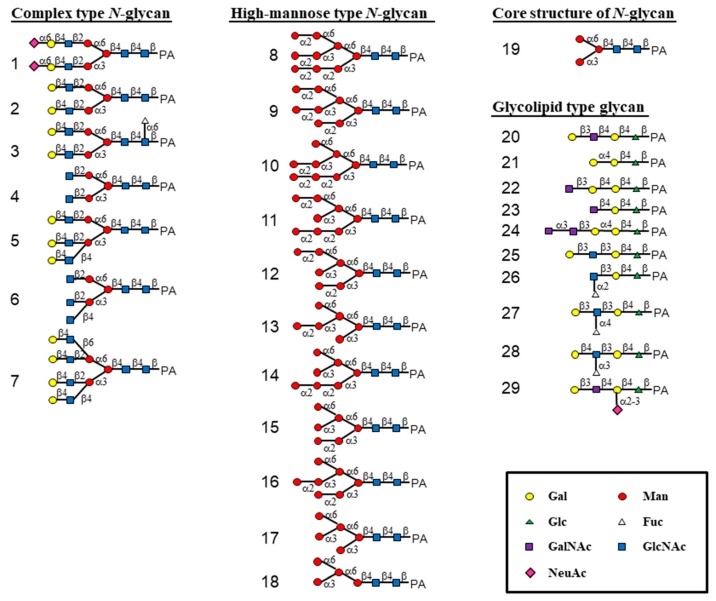
Schematic structures of 29 pyridylaminated (PA)-oligosaccharides used in this study. The selected oligosaccharides represent diverse carbohydrate structures; complex type *N*-glycans (**1**–**7**), high-mannose type *N*-glycans (**8**–**18**), an *N*-glycan core pentasaccharide (**19**), and oligosaccharides originated from glycolipid (**20**–**29**). Monosaccharide residues of Gal, Man, Glc, Fuc, GalNAc, GlcNAc and NeuAc are represented by symbols as shown in the box.

**Figure 3:**
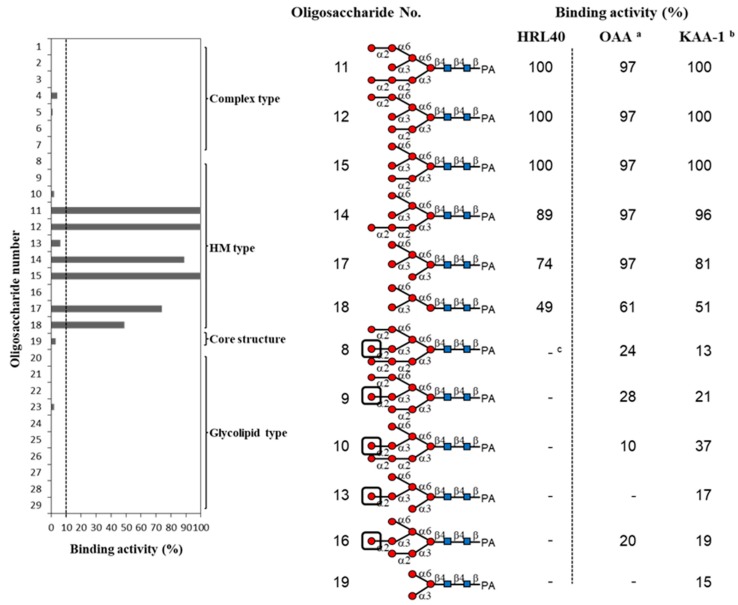
Binding activity of HRL40 to PA-oligosaccharides. The oligosaccharide-binding activity was determined by a centrifugal ultrafiltration-HPLC method [6,28]. The binding activity was expressed as a ratio (%) of the amount of bound PA-oligosaccharide [O_bound_] to that added [O_added_], where the [O_bound_] was obtained by subtracting the amount of unbound PA-oligosaccharide [O_unbound_] from [O_added_]. The [O_unbound_] was quantified by reverse-phase HPLC as described in the Materials and Methods section. The experiments were performed in triplicate for a PA-oligosaccharide and the activity was obtained as the average value. Black boxes represent the non-reducing terminal α1-2 linked mannose residue in the D2 arm. In this assay, the activities less than 10% were cut off for their insignificance. ^a^ data were cited from Sato et al. [15]. ^b^ data of His-tagged recombinant *Kappaphycus alvarezii* lectin (KAA-1 )were cited from Hirayama et al. [12]. ^c^ indicates the binding activity less than 10%.

The oligosaccharide-binding property of HRL40 was examined by a centrifugal ultrafiltration-HPLC method [6,28] with a serial of pyridylaminated (PA-) oligosaccharides, including complex type *N*-glycans (**1**–**7** in Figure 2 and Figure 3), HM-glycans (**8**–**18**), an *N*-glycan core pentasaccharide (**19**), and oligosaccharides originated from glycolipids (**20**–**29**). The schematic structures of oligosaccharides examined are shown in Figure 2. In the assay, the binding activity was expressed as the ratio (%) of the amount of a bound PA-oligosaccharide to that of an added PA-oligosaccharide.

As shown in Figure 3, HRL40 exclusively bound to some of HM-glycans (**11**, **12**, **14**, **15**, **17** and **18**), and had no binding interaction with other sugar types; complex type *N*-glycans (**1**–**7**), an *N*-glycan core pentasaccharide (**19**), and oligosaccharides from glycolipids (**19**–**29**). The binding preference of HRL40 for HM-glycans depended on the structure of branched oligomannosides; the highest binding activity was observed with oligosaccharides **11**, **12** and **15** (binding activity, 100%), a little less binding activity with oligosaccharides **14** (89%) and **17** (74%), and moderate activity was with an oligosaccharide **18** (49%). Thus, the HM-glycans recognized by HRL40 possessed in common an exposed (α1-3) mannose (Man) residue in the D2 arm of branched mannosides. On the other hand, no interaction was observed with HM-oligosaccharides **8**, **9**, **10**, **13** and **16,** which have a non-reducing terminal (α1-2) Man attached to the (α1-3) Man residue in the D2 arm. These results clearly indicated that HRL40 specifically recognized HM-glycans bearing an exposed (α1-3) Man residue in the D2 arm and that the binding affinity could be impaired by the addition of (α1-2) Man to the (α1-3) Man residue. Otherwise, the presence of the (α1-3) Man arm of a trimannosyl core, as well as the (α1-2) Man attached to the (α1-3) Man residue on the trimannosyl core, appeared to enhance the binding affinity because the lack of the residues lowered the binding activity, as compared between oligosaccharides **15** (100%) and **17** (74%), and between **17** and **18** (49%). The carbohydrate-binding profile resembled those of Type I HM-specific algal lectins (or the OAA family), such as the lectins from a blue green alga (cyanobacterium) *O. agardhii* (OAA) [15] and a red alga *K. alvarezii* (KAA-1) [12] as shown in Figure 3.

### 2.4. Inhibition of Virus Infection by HRL40

**Figure 4:**
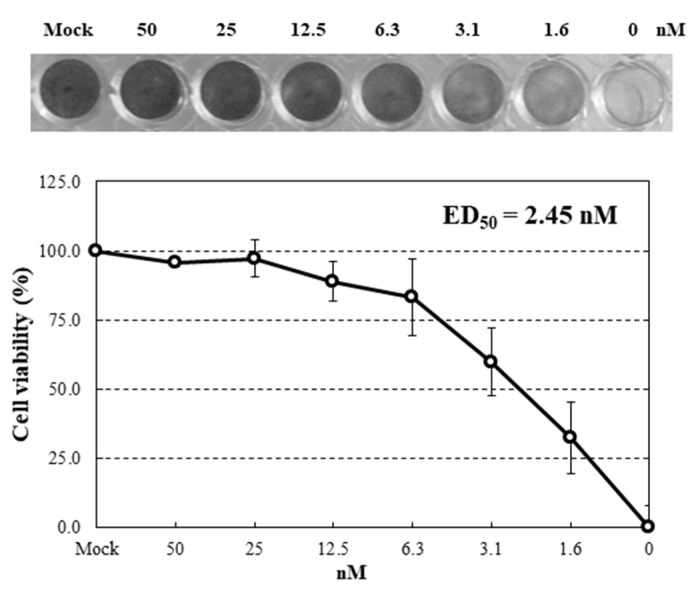
Dose-dependent anti-influenza virus activity of HRL40. NCI-H292 cells grown in 48-well plate were infected with influenza virus A/H3N2/Udorn/72 strain at a multiplicity of infection (m.o.i.) of 2.5 in the presence or absence of serial diluted lectins. At 24 h post-infection (hpi), the infected cells were fixed with 80% acetone and stained with amide black.

To examine anti-influenza activity of HRL40, infections with an influenza virus A strain A/H3N2/Udorn/72 were performed using human NCI-H292 cells in the presence of serial diluted concentrations of lectin. Under these infections with the virus strain in the absence of the lectins, almost all cells were dead at 24 h post-infection (hpi). Dose-effects of HRL40 on the inhibition of infection were investigated (Figure 4). This lectin was demonstrated to be potently active against the influenza virus. The extent of the infection-inhibition correlated with the increased concentrations of HRL40 in the cultures. The lectin concentration giving 50% inhibition of infection (ED_50_) was obtained by reading the point at which the curve crossed the 50% line. The ED_50_ for HRL40 was 2.45 nM (Figure 4), suggesting that this lectin might be a promising antiviral agent for its striking inhibitory ability of virus infection.

### 2.5. Interaction between HRL40 and Influenza Virus Envelope Hemagglutinin 

**Figure 5:**
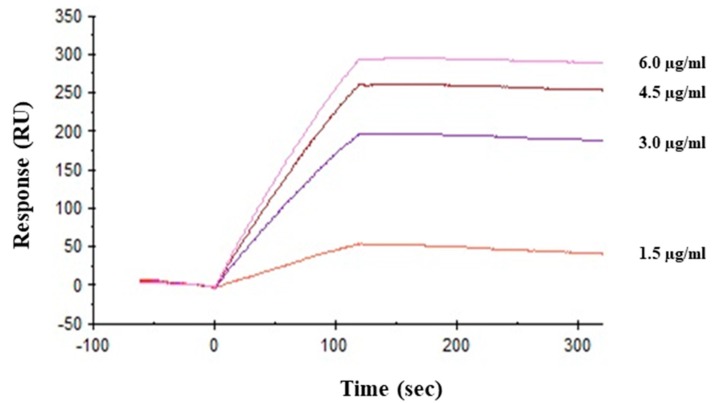
Interaction between HRL40 and influenza virus glycoprotein hemagglutinin was analyzed by surface plasmon resonance (SPR) using a Biacore X100 system (GE Healthcare Bio-Sciences AB, Uppsala, Sweden).

Direct interaction between viral hemagglutinin and HRL40 was tested by surface plasmon resonance (SPR) analysis. Hemagglutinin was immobilized onto the sensor chip (CM5) as the amount corresponding to 9672.6 resonance units (RU). As shown in Figure 5, lectin directly bound to hemagglutinin in a dose dependent manner and the affinity of HRL40 for hemagglutinin was revealed by the K_D_ value of 3.69 × 10^−11^ M (Table 3).

### 2.6. Molecular Structure of HRL40

**Figure 6:**
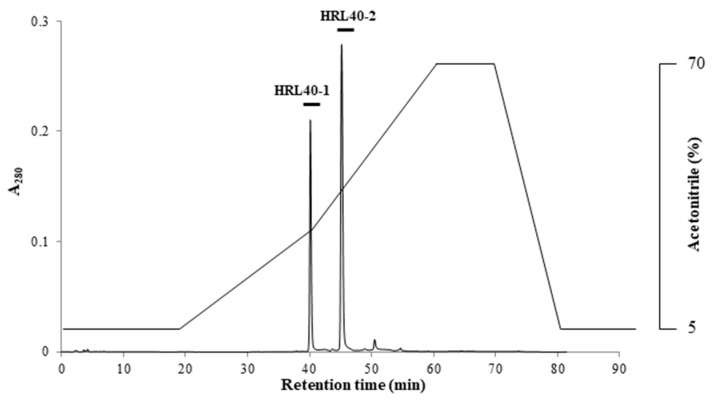
Reverse-phase HPLC of HRL40. Reverse-phase HPLC was performed on a TSKgel ODS-80TM combined with a gradient elution of acetonitrile in 0.05% trifluoroacetic acid (TFA).

**Figure 7:**
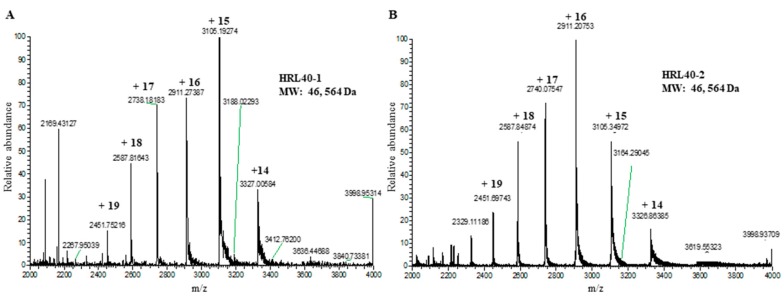
Electrospray ionization-mass spectrometry (ESI-MS) of HRL40-1 (**A**) and HRL40-2 (**B**). The molecular weights of both isolectins were obtained as deconvolution masses.

The relative molecular weight of the purified lectin HRL40 was determined to be about 40 kDa and 10 kDa in non-reducing and reducing SDS-PAGE, respectively (Figure 1D), which suggested that the lectin is an SS-linked tetrameric protein consisting of a 10 kDa polypeptide. Meanwhile, HRL40 was further separated into two isolectins (HRL40-1 and HRL40-2) when it was subjected to reverse-phase HPLC on a TSKgel ODS-80TM column combined with a gradient elution of acetonitrile in 0.05% trifluoroacetic acid (TFA) (Figure 6). In the analyses by electrospray-ionization mass spectrometry (ESI-MS), HRL40-1 and HRL40-2 showed the same molecular weight of 46,564 Da (Figure 7), suggesting that they are isoforms to each other.

**Figure 8:**
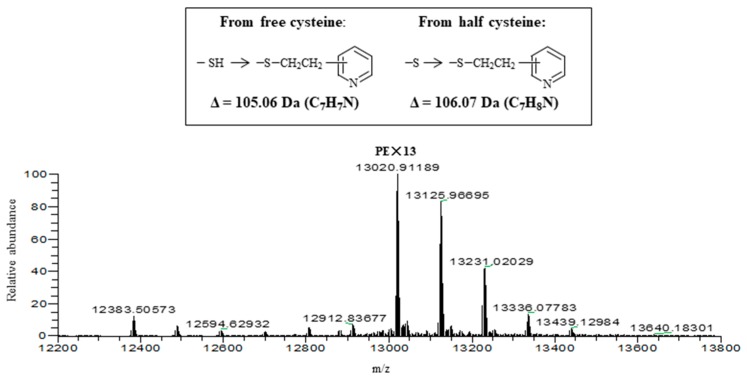
Electrospray ionization-mass spectrometry (ESI-MS) of *S*-pyridylethylated HRL40-1 in the presence of a reducing agent. HRL40-1 (6 µg) was subjected to reduction and alkylation in the presence of 0.11 M tri-n-butylphosphine.

Both isolectins were subjected to alkylation with 4-vinylpyridine in the presence of a reducing agent, tri-n-butylphosphine, and then applied to liquid chromatography (LC)-ESI-MS. As shown in Figure 8, where the result with pyridylethylated (PE-) HRL40-1 is represented, the multiple deconvolution mass of 13,020 Da was observed along with additional larger masses. The mass of 13,020 Da can be attributed to the PE-derivatives at 13 half-cystine residues of a subunit polypeptide (11,641 Da) of the SS-linked tetrameric lectin protein (46,564 Da) because the difference mass of 1379 Da (13,020–11,641 Da) corresponds to 13 times of the increased mass (+106 Da) by the addition of a pyridylethyl group at a reduced half-cystine. On the other hand, the masses larger than 13,020 Da may be derived from pyridylethylation at free cysteine residues where the mass increase by 105 Da each was observed. This estimation was also supported by the comparison of molecular weights of intact HRL40-1 and *S*-pyridylethylated HRL40-1 in the absence of a reducing agent (Figure S1). Thus, this lectin contained a lot of disulfide bonds in the molecule. HRL40-2 was also analyzed in a similar way and almost the same results were obtained (Figure S2). Thus, the protein structures of HRL40-1 and HRL40-2 appear to be quite different from those of Type I HM-specific algal lectins (the OAA family), which are monomeric proteins and have one or no cysteine residues in the molecule [6,10,15,29].

HRL40-1 and HRL40-2 were examined for their *N*-terminal sequences by a protein sequencer (Procise 492-HT, Applied Biosystems, Foster, CA, USA). However, the *N*-terminal amino acids cannot be sequenced, probably due to the modification at their *N*-termini. The treatment with 6 N HCl followed by the enzyme digestion with Pfu pyroglutamate aminopeptidase for deblocking formyl and pyroglutamyl groups were not effective in determining the *N*-terminal amino acids of both isolectins.

## 3. Discussion

HM-binding lectins have been attracting attention because they showed potent anti-viral activities by inhibiting the entry of viruses to the target host cells through binding with the viral envelope glycoproteins containing HM-glycans. We recently discovered and characterized HM-binding lectins from lower organisms, including marine algae and a blue-green alga (cyanobacterium) [6,8,9,10,12,15]. They were strictly specific for HM-glycans and most of them have no affinity for monosaccharides, including mannose and are thermostable monomeric proteins. We demonstrated that these HM-specific lectins thus far isolated by our group can be subdivided into four families (Types I–IV) based on their primary structures and recognition modes of branched mannosides [30]. Among them, Type I lectins are specific for HMs bearing an exposed (α1-3) Man in the D2 arm, and their binding activities are remarkably decreased with those having a non-reducing terminal (α1-2) Man attached to the (α1-3) Man residue. This group includes the lectins from several red algal species, such as *E. serra* (ESA) [6], *K. alvarezii* (KAA) [9,12], *K. striatum* (KSA) [10] and a blue-green alga (cyanobacterium) *O. agardhii* (OAA) [15]. From the cDNA cloning of a cyanobacterial lectin, OAA, we presented that the genes coding Type I lectin proteins are widely distributed in lower organisms [6,12,29], including red algae and bacteria such as *Myxococcus xanthus* hemagglutinin (MBHA) [31], *Pseudomonas fluorescens* lectin (PFL) [32] and *Burkholderia oklahomensis* agglutinin (BOA) [33]. MBHA and PFL were confirmed to inhibit the HIV-infection in the host cells using their recombinants [34,35]. The Type I HM-specific lectins (the OAA family) consist of two or four tandem repeats of the conserved domain of 66–67 amino acids. The crystal structures and NMR analyses indicated that the monomeric lectins of this family had the plural glycan binding sites in a polypeptide chain, in which two and four binding sites were formed by swapping between two and four tandemly repeated domains, respectively [36,37]. The amino acid sequences were well conserved between domains as well as between the lectin species within this family [6,12,29]. In *in silico* search on GenBank database, the similar sequences have not been found in any proteins from animals, higher plants and green algae (Chlorophyta). 

In this study, we isolated an HM-specific lectin, named HRL40 from the calcareous marine alga, *H. renschii*, for the first time. In terms of the oligosaccharide-binding specificity, HRL40 clearly belongs to the OAA family (Type I) because this lectin strictly recognized the HM-glycans having an exposed (α1-3) Man in the D2 arm (Figure 3). Thus, HRL40 is the first Type I HM-specific lectin from the Chlorophyta. As seen in Figure 3, the binding preference of HRL40 for the exposed (α1-3) Man residue was markedly severe among Type I lectin family, promising the utility of this lectin as a valuable tool in the fields of basic and applied glycomics. To be exact, HRL40 potently inhibited the entry of influenza virus (A/H3N2/Udorn/72) to NCI-H292 cells with ED_50_ of 2.45 nM, which was the highest anti-influenza virus activity that we have ever examined for lectins [8,9,13,35]. In addition, the affinity constant (K_D_, 3.69 × 10^−11^ M) of this lectin for the influenza virus envelope hemagglutinin was also remarkably high. Depending on the viral strain, the influenza viral hemagglutinins contain the HM-glycan(s) in the molecule, which is/are located close to the binding site for *N*-acetylneuraminic acid on the surface receptor of the target host cells [23]. It is therefore supposed that HRL40 inhibited the viral entry through binding to the HM-glycan of the viral hemagglutinin. 

Despite HRL40 showing typical carbohydrate binding profile of Type I HM-specific lectins, its molecular structure was quite different from the family. HRL40 was an SS-linked tetrameric protein containing more than 13 half-cystine residues, in contrast to other lectins of the OAA family, which are monomeric and have one or no cysteine residues in the molecule. In addition, the presence of free cysteine residues was also suggested for this lectin (Figure S1). We preliminarily prepared the peptide fragments of HRL40-1 using a few kinds of protease after *S*-pyridylethylation under a reducing agent and checked their sequences by nanoLC-MS/MS, and detected a lot of peptide fragments containing *S*-pyridylethyl-modified sequences. However, the peptide fragments having the sequences similar to the OAA family were not searched out. HRL40-1 and HRL40-2 shared the identical molecular weights and the number of disulfide bonds, but nevertheless they were clearly separated by reverse-phase HPLC (Figure 6). It may be possible that both isolectins may differ in spatial structure or that they may have the substitution of a few amino acids, which does not affect the molecular weights. Due to the lack of available sequence information, including *N*-terminal amino acids, cDNA cloning of HRL40 remains to be clarified in the future.

## 4. Materials and Methods

### 4.1. Materials

The specimens of the green alga *H. renschii* were collected on the coast of Yakushima, Kagoshima Prefecture, Japan, in July, 2012. The collected sample was stored in a freezer at −30 °C until used. HiPrep Phenyl FF and Superdex-75 columns were purchased from GE Healthcare Bio-Sciences AB (Uppsala, Sweden), and TSKgel DEAE-5PW and TSKgel ODS-80TM columns were from Tosoh Co. (Tokyo, Japan). d-Glucose (Glc), d-galactose (Gal), *N*-acetyl-d-galactosamine (GalNAc), transferrin, fetuin, bovine submaxillary mucin (BSM), and porcine thyroglobulin (PTG) were purchased from Sigma-Aldrich Co. (St. Louis, MO, USA). d-Mannose (Man), l-fucose (Fuc), *N*-acetyl-d-glucosamine (GlcNAc), *N*-acetyl-d-neuraminic acid (NeuAc), lactose, and yeast mannan were obtained from Nacalai Tesque Co. (Kyoto, Japan). d-Xylose (Xyl) and l-rhamnose (Rha) were obtained from Wako Chemical Co. (Osaka, Japan). The desialylated (asialo-) derivatives of glycoproteins were prepared by hydrolysis of the parent sialoglycoproteins with 0.1 N HCl for 1 h at 80 °C as described by Hori et al. [38]. PA-oligosaccharides were purchased from Takara Bio (Tokyo, Japan). All other reagents used in this study were of the highest purity available. Influenza virus strain A/H3N2/Udorn/72 was kindly supplied by Takemasa Sakaguchi (Graduate School of Biomedical & Health Sciences, Hiroshima University, Hiroshima, Japan) and H292 cell (ATCC #CRL1848) was purchased from Culture Collections, Public Health England (London, UK).

### 4.2. Extraction and Purification of Lectin HRL40

A frozen sample of *H. renschii* (2 kg) was thawed and ground in liquid nitrogen to a powder. To the powdered alga, 2 volumes (*v*/*w*) of PBS was added and stirred at 4 °C overnight. The mixture was centrifuged at 9000 *g* for 30 min at 4 °C. To the recovered supernatant, solid ammonium sulfate was added to attain a 70% saturation. The mixture was gently stirred for 30 min and kept at 4 °C overnight. The precipitates were recovered after centrifugation (9000 *g*, 30 min, 4 °C), dissolved in a small volume of PBS, and dialyzed thoroughly against the same buffer. The inner fraction was further centrifuged (9000 *g*, 30 min, 4 °C) to remove the insoluble components generated during dialysis, and the supernatant was recovered as a salting-out fraction. The salting-out fraction was adjusted to 1 M solution with solid ammonium sulfate, and applied to a HiPrep Phenyl FF column (1.6 × 10 cm) equilibrated with 1 M ammonium sulfate in PB. The column was thoroughly washed with the starting solution and then eluted with PB. The flow rate was 2 mL/min. Fractions of 10 mL were collected and measured for absorbance at 280 nm (A_280_) and HA. Active fractions eluted with PB were pooled and concentrated by ultrafiltration (molecular weight cut off (MWCO) of 10 kDa). The concentrate was adjusted to 1 M solution with solid ammonium sulfate, and applied to the same HiPrep Phenyl FF column equilibrated with 1 M ammonium sulfate in PB. The column was washed with the same solution and then eluted by a linear gradient of ammonium sulfate (1.0–0 M) in PB. The elution was performed at a flow rate of 2 mL/min. Fractions of 5 mL were collected and measured for A_280_ and HA. Active fractions were pooled, concentrated by ultrafiltration, and subjected to gel-filtration on a Superdex-75 column (1.0 × 30 cm) equilibrated with PBS (pH 7.0) containing 0.3 M NaCl. After injection of the sample, the column was eluted with the same buffer and the eluate was monitored by A_280_. The peak fractions were manually collected and measured for HA. A major active peak was recovered, dialyzed against 20 mM Tris-HCl buffer (pH 8.0), and applied to a TSKgel DEAE-5PW column (7.5 × 75 mm) equilibrated with 20 mM Tris-HCl buffer (pH 8.0). The column was washed with the same buffer and then eluted with a linear gradient of NaCl (0–1 M) in the buffer. Fractions of 1 mL were collected and measured for A_280_ and HA.

### 4.3. Determination of Protein Content

Protein content was determined by A_280_, presuming that a protein concentration of 1 mg/mL gives A_280_ of 1.0, or by the Pierce BCA Protein Assay Kit (Thermo Fisher Scientific, Rockford, IL, USA) using bovine serum albumin as a standard. 

### 4.4. Sodium Dodecyl Sulfate-Polyacrylamide Gel Electrophoresis (SDS-PAGE)

SDS-PAGE was performed using a 12% polyacrylamide gel according to the method of Schägger and Jagow [39]. Samples were heated at 100 °C for 5 min in a loading buffer containing 0.2% SDS with or without 2% 2-mercaptoethanol. Electrophoresis was carried out at a constant voltage of 100 V for 2 h. After electrophoresis, the gel was stained with CBB R-250. A marker kit containing reference proteins was purchased from Tefco (Tokyo, Japan).

### 4.5. Hemagglutination and Hemagglutination-Inhibition Assays

Hemagglutination and hemagglutination-inhibition assays were performed using a 2% (*v*/*v*) suspension of trypsin or Pronase-treated rabbit erythrocytes as described by Hori et al. [40]. The rabbit blood was purchased from Hiroshima Animal Research Institute (Hiroshima, Japan). The following sugars and glycoproteins were tested in this study: as monosaccharides, Glc, Gal, Man, Fuc, GalNAc, GlcNAc, Xyl, Rha, and NeuAc; as a disaccharide, lactose; as glycoproteins, transferrin, asialo-transferrin, fetuin, asialo-fetuin, BSM, asialo-BSM, PTG, asialo-PTG, and yeast mannan. Inhibition was observed macroscopically, and inhibition activity was given as the minimum inhibition concentration of sugar (mM) or glycoprotein (μg/mL) at which complete inhibition of hemagglutination of lectin solution having the titer 4 was achieved.

### 4.6. Oligosaccharide-Binding Analysis

The oligosaccharide-binding specificity was determined by a centrifugal ultrafiltration-HPLC method as described by Hori et al. [6,28]. Briefly, 90 µL of 1 µM lectin solution and 10 µL of 300 nM PA-oligosaccharide were mixed in 50 mM Tri-HCl (pH 7.0) and incubated for 1 h at room temperature. The reaction mixture was centrifuged at 10,000 *g* for 30 s at room temperature using a centrifugal ultrafiltration device (Pall, Ann Arbor, MI, USA) (MWCO of 10 kDa). The unbound PA-oligosaccharide was recovered in the filtrate. To quantify the unbound PA-oligosaccharide, 20 µL of the filtrate was applied to a TSKgel ODS-80TM column (4.6 × 150 mm) equilibrated with 15% methanol in 0.1 M ammonium acetate buffer and eluted with the same buffer. The HPLC was performed at a flow rate of 1 mL/min at 40 °C. The eluate was monitored for PA-oligosaccharide at an excitation wavelength of 320 nm and an emission wavelength of 400 nm. Meanwhile, 90 μL of 50 mM Tris-HCl (pH 7.0) without lectin was mixed with the same PA-oligosaccharide, and the mixture was centrifuged as described above and the filtrate was used as a blank which represented the amount of added PA-oligosaccharides. The amount of bound PA-oligosaccharide [O_bound_] was obtained by subtracting the amount of unbound PA-oligosaccharide [O_unbound_] from that of added one [O_added_]. The binding activity was defined as the ratio of [O_bound_] to [O_added_] and denoted as % binding. The binding experiments were performed in triplicate for every PA-oligosaccharide and the activity was expressed as an average value.

### 4.7. Molecular Weight Determination of HRL40

Prior to MS analyses, the purified lectin HRL40 was subjected to reverse-phase HPLC on a TSKgel ODS-80TM column (4.6 × 150 mm) equilibrated with 5% acetonitrile in 0.05% aqueous TFA. After loading of HRL40, the column was washed with the starting solution and then eluted by a gradient of 5–70% acetonitrile in 0.05% TFA. The eluate was monitored by A_280_ and protein peaks were manually collected. The purified lectins, HRL40-1 and HRL40-2, thus obtained were used for molecular weight determination.

Mass analyses were carried out by LC-ESI-MS with an LTQ Orbitrap XL (Thermo Fisher Scientific). Sample was applied to a ZORBAX 300SB-C8 column (1 × 50 mm, 3.5 μm, Agilent, Santa Clara, CA, USA) equilibrated with 0.1% (*v*/*v*) aqueous formic acid (A) and eluted by a gradient between (A) and 100% acetonitrile in (A) at a flow rate of 80 µL/min. The tuning parameters used for mass analyses were as follows; capillary temperature 300 °C, source voltage 4.5 kV, capillary voltage 50 V and tube lens voltage 170 V.

### 4.8. Preparation of S-Pyridylethylated Lectins 

*S*-pyridylethylation was performed as previously described by Hori et al. [6]. Briefly, lectin protein (6 µg) was dissolved in 100 µL of 0.5 M Tris-HCl (pH 8.5) containing 6 M guanidine and 10 mM EDTA. The protein solution was mixed gently with 0.26 M 4-vinylpyridine in the presence or absence of a reducing reagent, 0.11 M tri-n-butylphosphine. The reaction mixture was incubated at room temperature overnight in a dark room and then desalted on a Pierce C18 Spin column (Thermo Fisher Scientific) where PE-lectin protein was eluted by 70% acetonitrile in 0.1% TFA. The purified PE-lectin protein was dried and kept at −20 °C for further experiments.

### 4.9. Anti-Influenza Activity Test

NCI-H292 cells grown in 48-well plate were infected with influenza virus A/H3N2/Udorn/72 strain at a multiplicity of infection (m.o.i.) of 2.5. Different concentrations of the lectin were added simultaneously into the cell cultures. At 24 hpi, the infected cells were fixed with 80% acetone, and stained with 0.5% amide black in 45% ethanol and 10% acetic acid. The stained plates were pictured with a gray scale. The color densities of the pictures were quantitated by densitometry with the NIH-ImageJ 1.48 v software (National Institutes of Health, Bethesda, MD, USA) as described elsewhere [35]. The infected cell cultures in the absence of lectin exhibited severe cytopathic effect, almost all cells on the wells were gone, in which percent of cell viability was shown as 0%. On the other hand, cells in the mock-infected cell cultures were intact, in which percent of the cell viability was shown as 100%.

### 4.10. Surface Plasmon Resonance Analysis of Interaction between HRL40 and Influenza Viral Hemagglutinin

Direct interaction of HRL40 with the influenza viral envelope glycoprotein hemagglutinin was analyzed on a Biacore X100 system. The sensor chip (CM5, GE Healthcare Bio-Sciences AB) was activated with *N*-hydroxysuccinimide/*N*-ethyl-*N*′-dimethylaminopropyl carbodiimide, and hemagglutinin was immobilized onto the sensor chip by amine coupling method. The unreacted groups on the sensor surface were blocked with 1 M ethanolamine. Binding experiments were performed using various concentrations of lectins at a flow rate of 30 μL/min with a running buffer (HBS-N) consisting of 10 mM HEPES, 150 mM NaCl (pH 7.4). The condition of kinetics/affinity assay was as follows: contact time, 120 s, dissociation time, 600 s. The surface was regenerated by 10 mM glycine-HCl (pH 1.5). Kinetic parameters (k_a_, k_d_, K_A_ and K_D_) were calculated by fitting the data to the Langmuir model for 1:1 binding using the Biacore X100 evaluation software (GE Healthcare Bio-Sciences AB).

## 5. Conclusions

A novel lectin was isolated from the green alga *H. renschii*. The lectin was strictly specific for HM-glycans having an exposed (α1-3) mannose in the D2 arm of branched mannoside and showed potent anti-influenza virus activity through strong binding to the viral envelope hemagglutinin. The biological properties of this lectin resembled those of Type I HM-specific lectins from lower organisms (the OAA family). However, its molecular structure, which is an SS-linked tetrameric protein of a 11,641 Da polypeptide containing at least 13 half-cystines, was quite different from the family.

## Figures and Tables

**Table 1:** Purification of a lectin (HRL40) from *H. renschii*.

Purification Procedure	Volume (mL)	Protein ^a^ (mg/mL)	HA ^b^	THA ^c^	MAC ^d^ (μg/mL)
Extraction	4000	7.0	32	128,000	218.8
Salting-out	100	20.0	1024	102,400	19.5
HP ^e^ (stepwise)	94	3.2	128	12,032	25.0
HP (gradient)	75	1.6	64	4800	25.0
GF ^f^	26	0.5	64	1664	7.8
IE ^g^	1	1.3	64	64	20.3

^a^ Protein concentration was estimated by A_280_ on the assumption that A_280_ at 1.0 mg/mL is 1.0; ^b^ HA, hemagglutination activity (titer) determined with trypsin-treated rabbit erythrocytes; ^c^ THA, total hemagglutination activity (HA × volume); ^d^ MAC, minimum agglutination concentration, the protein concentration of the highest dilution exhibiting positive hemagglutination; ^e^ HP, hydrophobic chromatography; ^f^ GF, gel-filtration chromatography; ^g^ IE, ion-exchange chromatography.

**Table 2:** Hemagglutination-inhibition profiles of active fractions and purified HRL40 with sugar compounds.

Sugars and Glycoproteins ^a^	Salting-Out	HP ^b^ (Stepwise)	HP (Gradient)	GF ^c^	HRL40
Sugars (mM)					
Monosaccharides ^d^	>100	>100	>100	>100	>100
Disaccharide					
Lactose	>100	>100	>100	>100	>100
Glycoproteins (μg/mL)					
*N*-glycan-linked					
Complex type					
Transferrin	>1000	>1000	>1000	>1000	>1000
Asialo-transferrin	>1000	>1000	>1000	>1000	>1000
High-mannose type					
Yeast mannan	>1000	>1000	31.3	31.3	15.6
Complex and high-mannose types					
Porcine thyroglobulin (PTG)	250	250	62.5	125	62.5
Asialo-PTG	250	500	250	500	250
*N/O*-glycan-linked					
Fetuin	>1000	>1000	1000	>1000	>1000
Asialo-fetuin	>1000	>1000	>1000	>1000	1000
*O*-glycan-linked					
Bovine submaxillary mucin (BSM)	>1000	>1000	1000	>1000	1000
Asialo-BSM	>1000	>1000	1000	1000	1000

^a^ Values indicate the lowest concentration of sugars (mM) and glycoproteins (μg/mL) that completely inhibited the hemagglutination of the lectin solution of a titer, 4. In the hemagglutination-inhibition test, a 2% suspension of Pronase-treated rabbit blood cells was used; ^b^ HP, hydrophobic chromatography; ^c^ GF, gel-filtration chromatography; ^d^ d-Glucose (Glc), d-mannose (Man), d-galactose (Gal), *N*-acetyl-d-galactosamine (GalNAc), *N*-acetyl-d-glucosamine (GlcNAc), l-fucose (Fuc), d-xylose (Xyl), l-rhamnose (Rha), and *N*-acetyl-d-neuraminic acid (NeuAc).

**Table 3:** Binding kinetics of the interaction between HRL40 and influenza virus hemagglutinin.

	k_a_ (M^−1^s^−1^)	k_d_ (s^−1^)	K_A_ (M^−1^)	K_D_ (M)
HRL40	7.14 × 10^4^	2.63 × 10^−6^	2.71 × 10^10^	3.69 × 10^−11^

k_a_, association rate constant; k_d_, dissociation rate constant; K_A_, association constant; K_D_, dissociation constant.

## Supplementary Materials

The following materials are available online at www.mdpi.com/1660-3397/15/8/255/s1, Figure S1: Electrospray ionization-mass spectrometry (ESI-MS) of intact HRL40-1 and HRL40-2 and their *S*-pyridylethylated derivatives in the absence of a reducing agent. Figure S2: Electrospray ionization-mass spectrometry (ESI-MS) of *S*-pyridylethylated HRL40-2 in the presence of a reducing agent (tri-n-butylphosphine) (B).
