# Supplementary material for: A Novel High-Mannose Specific Lectin from the Green Alga Halimeda renschii Exhibits a Potent Anti-Influenza Virus Activity through High-Affinity Binding to the Viral Hemagglutinin
Source: Mar Drugs. 2017 Aug 16;15(8):255. doi: 10.3390/md15080255 (PMC5577609; doi:10.3390/md15080255)
Supplement: Supplementary file 1 [file marinedrugs-15-00255-s001.pdf]

# Supplementary Materials: A Novel High-Mannose Specific Lectin from the Green Alga *Halimeda renschii* Exhibits a Potent Anti-Influenza Virus Activity through High-Affinity Binding to the Viral Hemagglutinin

**Jinmin Mu, Makoto Hirayama, Yuichiro Sato, Kinjiro Morimoto and Kanji Hori**

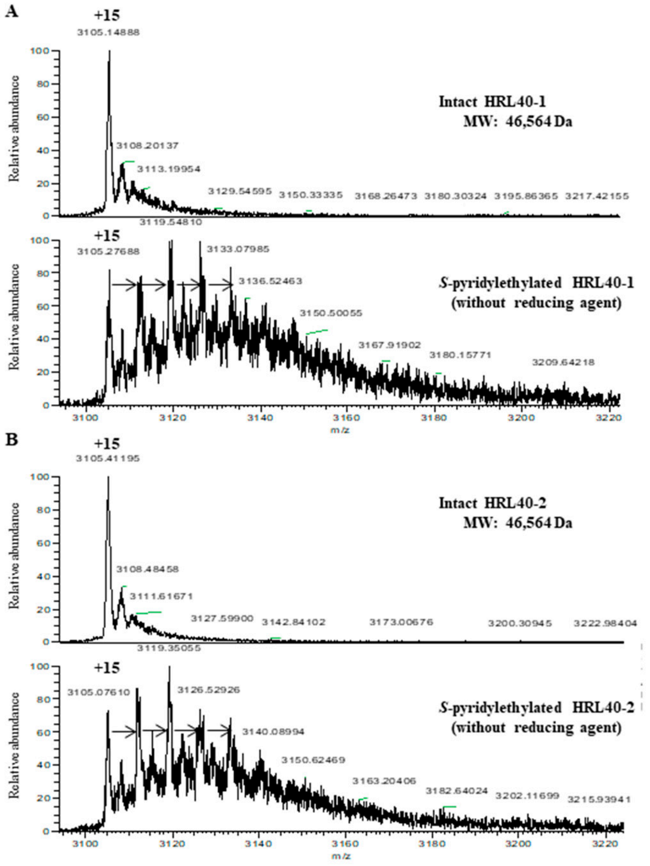


**Figure S1.** Electrospray ionization-mass spectrometry (ESI-MS) of intact HRL40-1 and HRL40-2 and their *S*-pyridylethylated derivatives in the absence of a reducing agent.

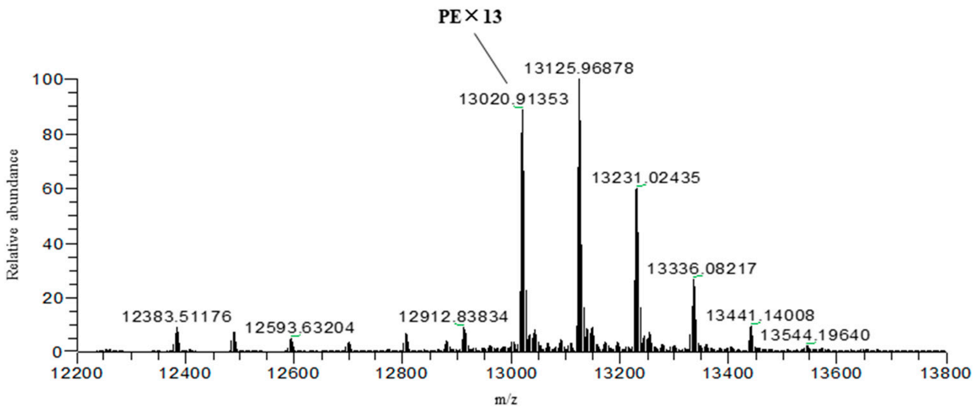


**Figure S2.** Electrospray ionization-mass spectrometry (ESI-MS) of *S*-pyridylethylated HRL40-2 in the presence of a reducing agent (tri-n-butylphosphine) (B).
